# Human Young Children as well as Adults Demonstrate ‘Superior’ Rapid Snake Detection When Typical Striking Posture Is Displayed by the Snake

**DOI:** 10.1371/journal.pone.0015122

**Published:** 2010-11-30

**Authors:** Nobuo Masataka, Sachiko Hayakawa, Nobuyuki Kawai

**Affiliations:** 1 Primate Research Institute, Kyoto University, Inuyama, Japan; 2 Graduate School of Information Science, Nagoya University, Nagoya, Japan; University of Western Ontario, Canada

## Abstract

Humans as well as some nonhuman primates have an evolved predisposition to associate snakes with fear by detecting their presence as fear-relevant stimuli more rapidly than fear-irrelevant ones. In the present experiment, a total of 74 of 3- to 4-year-old children and adults were asked to find a single target black-and-white photo of a snake among an array of eight black-and-white photos of flowers as distracters. As target stimuli, we prepared two groups of snake photos, one in which a typical striking posture was displayed by a snake and the other in which a resting snake was shown. When reaction time to find the snake photo was compared between these two types of the stimuli, its mean value was found to be significantly smaller for the photos of snakes displaying striking posture than for the photos of resting snakes in both the adults and children. These findings suggest the possibility that the human perceptual bias for snakes per se could be differentiated according to the difference of the degree to which their presence acts as a fear-relevant stimulus.

## Introduction

To react defensively in fearful situations has been crucial for the survival of all animal species. No doubt this is also true for humans (*Homo sapiense*) as a primate species. Most non-human primates are known to have evolved an innate predisposition to quickly associate fear with some specific threatening stimuli. This is typically the case for their response to poisonous snakes. In humans, too, snake-phobia is regarded as a phenomenon which is widespread throughout the world [1], [2]. An even stronger version of such an argument was recently published [3]. In the comprehensive analysis of the origin of the human visual system, the author discussed that some of its basic properties evolved precisely because they facilitated the detection of snakes. Evidence to support that argument included a series of investigations that showed that human adults have an attentional bias for the detection of fear-relevant stimuli such as snakes compared to neutral stimuli such as flowers and mushrooms [2], [4]. More recent studies have documented that preschool children, 8- to 14-month-old infants, and even non-human primates also detect snakes more quickly than flowers [5], [6], [7].

The results of that series of studies could serve as somewhat convincing evidence for the notion that humans have an evolved predisposition to associate snakes with fear by detecting their presence as fear-relevant stimuli more rapidly than fear-irrelevant ones. The present study was undertaken to extend these findings into an exploration of the possibility that such a bias toward snakes per se could be differentiated in humans according to the difference of the degree to which their presence acts as a fear-relevant stimulus. We reasoned that humans might be predisposed to respond to the presence of a snake more rapidly if circumstances where the humans are exposed to the snake are potentially more urgently threatening for their survival. In order to simulate such contextual variability, we prepared two groups of snake photos, one in which a so-called typical striking posture [8], [9] was displayed by a snake and the other in which a resting snake was photographed. In each experimental trial, a photo from either of these groups was chosen as a target stimulus, and presented with eight photos of flowers as distracters. We attempted to examine whether attentional responsiveness was affected or not by such variability, comparing how rapidly humans detected the target between the two stimulus groups.

## Methods

This investigation was conducted according to the principles expressed in the Declaration of Helsinki. All experimental protocols are consistent with the Guide for the Experimentation with Humans and were approved by the Institutional Ethical Committee of Primate Research Institute, Kyoto University. We obtained written informed consent from all participants involved in our study.

Twenty 3-year-old children (mean±SD = 44.5±2.6 months, range = 38–47), 34 4-year-old children (53.9±3.5 months, range = 48–59), and 20 adults (397.8±76.2 months, range = 296–522) participated in the experiments. According to parental reports, none of the children had ever experienced exposure to real or toy snakes, or any images of a snake prior to the experiment. None of the participants had visual or hearing impairments. Four additional children (two 3-year-olds and two 4-year-olds) who failed to follow directions were excluded.

**Figure 1:**
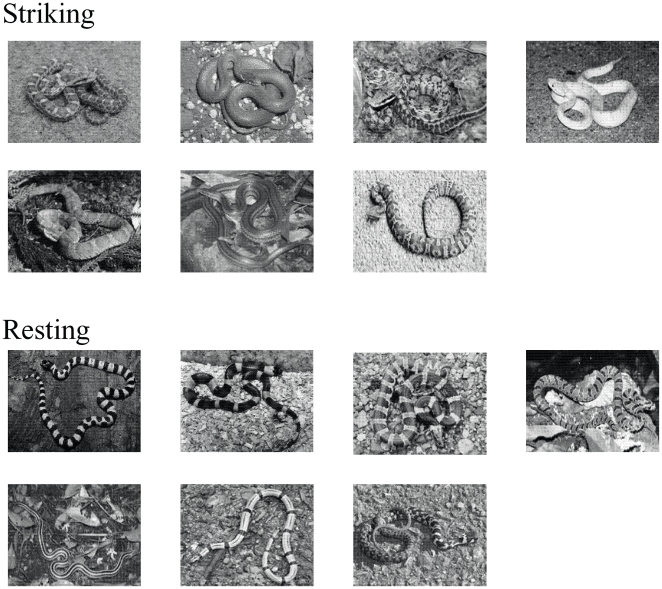
The 14 photographs of snakes which were used as target stimuli. In 7 of them, a typical striking posture was displayed (Striking) while a resting snake was displayed in the remaining 7 (Resting).

For the experiment, we selected 14 black-and white photographs for each of snake and flower categories. In each snake photo, a wild snake was photographed under the horizontal background of the natural substrate (Figure 1). The photo of the whole figure of the snake was taken into the frame using a camera that was positioned at a height of approximately 1.6 m, at a roughly 70– to 75-degree angle towards the target snake in relation to the horizontal plane. All the snakes were roughly uniform in body size, and 1.2 to 1.3 m in length. However, half of the 14 displayed a typical striking posture, with the body coiled and the neck held in an S-curve, a single segment of the body elevated, and the head poised to strike. They are referred to as the Stimulus Group of Striking Posture below. The snakes photographed in the other half of the 14 were resting, extending their entire bodies. They are referred to as the Stimulus Group of Resting Posture below.

**Figure 2:**
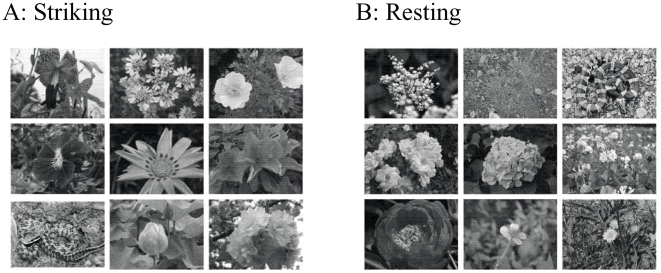
An example of a 3×3 matrix used as the stimulus in an experimental trial where a photo of striking posture of a snake was included (Striking), and one where a photo of a resting snake was included (Resting).

In a given trial, 9 of these 28 photographs were displayed in a 3×3 matrix (Figure 2). Each matrix contained 1 target photo from one category and 8 distracter photos from the other category. A color touch-screen monitor (RDT151TU, Mitsubishi, Japan) was used to present each picture matrix on a 38.1-cm (15-inch) screen. Each of the 14 photos in the target category served as the target once. Each of the 14 photos in the distracter category appeared multiple times; the different distracters were presented approximately the same number of times across trials. An outline of the participant's handprints was located on the table immediately in front of the monitor.

Each participant was seated in front of the touch-screen monitor (approximately 40 cm from the base of the screen) and was instructed to place his or her hands on the handprints (Figure 2) to ensure that the hands were in the same place at the start of each trial, making it possible to collect reliable latency data. An investigator was seated alongside the monitor and instructed the participant throughout the procedure. First, a set of 9 practice trials was given to teach the participant how to use the touch screen. In the first 3 trials, a display of 1 target (an animated puppet that is well-known among Japanese children) and 8 distracter (another well-known puppet) photos was presented. The participant was asked to touch the target among the distracters as quickly as possible, and then return his or her hands to the handprints. On the next 6 trials, the display consisted of 1 target (a snake or a flower) and 8 distracter (a flower when the target was a snake, and vice versa) photos, and the participant was asked to touch only the target photo. All snake photos used in the practice trials were chosen randomly from the original set of 14.

When the participants had adequately learned the procedure, a series of test trials followed. The task comprised 28 trials in total, ordered in two blocks of 14 trials. In each trial, a different photo matrix containing 1 target (snake or flower) and 8 distracters (as described above) was presented. Between trials, a photo of a stuffed animal or a popular character appeared on the screen to keep the participant's attention on the screen. The investigator initiated the next trial when she judged that the participant was looking at the photos, ensuring that the next matrix appeared so that the participant's full attention was on the screen. When the first block was over, another block began. If the first block target was snakes, the next target was flowers, or vice versa. Each participant was randomly assigned to one of two block orders.

In each trial, the reaction time (RT) of the participant was automatically recorded from the onset of the matrix to when the participant touched one of the photos on the screen. The results reported here were based solely upon analyses on the RT data collected in this manner (RTs of incorrect responses as well as extreme RT scores—defined as values more than two standard deviations above or below the mean relative to each participant's mean RT—were excluded from the analyses).

## Results

When a snake photo was presented as a target, overall mean (SD) RT was 2735 (686), 2439 (742), and 1060 (772) ms for the 3-year-olds, 4-year-olds and adults, respectively, while mean (SD) RT when a flower photo was presented was 3283 (599), 3232 (1144), and 1380 (442) ms for the 3-year-olds, 4-year-olds and adults, respectively. Both of the two main effects were statistically significant (*F* = (1,71) = 41.0 *p*<0.001 *η* ^2^_p_ = 0.376 for STIMULUS, *F* = (2,71) = 51.7 *p*<0.001 *η* ^2^_p_ = 0.603 for AGE). Interaction between the main factors was not significant (*F* = 2,71) = 1.95 *p* = 0.15).

**Figure 3:**
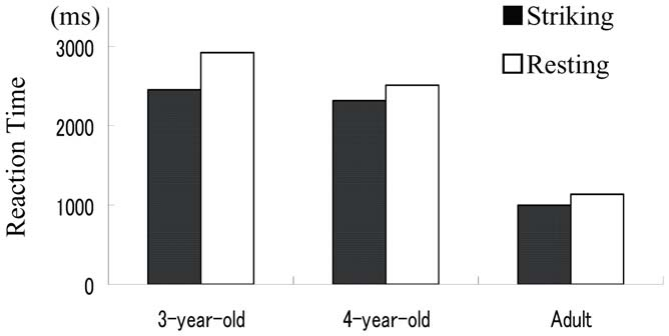
Mean reaction time to detect a snake when striking posture was displayed in the target photo and when a resting snake was shown in the target photo.

Next, when the data collected when one of the 28 snake photos was used as a target stimulus were compared between the two Stimulus Groups, both of the two main effects were statistically significant (*F* = (1,36) = 4.72 *p* = 0.036 *η* ^2^_p_ = 0.429 for STIMULUS, *F* = (2,36) = 73.6 *p*<0.001 *η* ^2^_p_ = 0.532 for AGE). However, interaction between the main factors was not significant (*F* = 2,36) = 0.92 *p* = 0.41). Mean (SD) RT when a snake photo of the Stimulus Group of Resting Posture was presented as a target was 2930 (392), 2519 (399), and 1097 (430) ms for the 3-year-olds, 4-year-olds and adults, respectively, while mean (SD) RT when a snake photo of the Stimulus Group of Striking Posture was presented was 2452 (428), 2321 (432), and 1000 (310) ms for the 3-year-olds, 4-year-olds and adults, respectively (Figure 3).

## Discussion

When aroused, snakes commonly attempt to strike rapidly forward with the mouth open. This behavior may be followed by an actual bite. Display of “striking posture” has been noted as the behavior preceding such attack in numerous reports (see [8.9] for review). In addition to confirming previous findings [2], [4], [5], [7], the present study clearly showed that humans detect snakes more rapidly in photos in which the snake displays a striking posture than in photos showing a resting snake.

A predispositional tendency of humans to associate physical attributes of external objects with some specific emotion, per se, is not a totally new notion. Concerning social perception, the fact has been known since the 1940s that adults are predisposed to associate a positive affective feeling with some physical design features of conspecific infants and young children, which in turn results in caregiving behavior by the adults [10]. The physical characteristics Lorenz documented include a protruding forehead, large eyes, rounded body shape, etc. A series of snake detection experiments [4], [5], [11] were apparently conceived as an extension of that direction of research [12], [13].

There are obvious functional implications of the evolution of such predisposition with respect to phobic behavior. Nevertheless, it should be noted that virtually nothing has been reported so far regarding which design features of snakes are important for the rapid detection of their presence. This is partly because in previous research about object recognition in humans, the general consensus has been that structural (shape) features are the primary mental representations, whereas surface characteristics play a role only when shape information is uninformative [14]. The sinusoidal pattern in unique to snakes among natural objects, and thus potentially prominent and reliable as a perceptual cue for rapid detection. As a result, structural representations of snakes as objects can be easily used for the primary access for their recognition [15] while a category such as ‘flowers’ refers not only to a prototypical image with a given structural description but also to many alternative images [16].

Concerning variation of the appearances of the snakes in the photos used in the present study, it should be noted that four of the seven resting snakes were clearly banded but none of the striking snakes were banded. Actually, all of the four are venomous snakes whose appearances have been referred to as a ‘warning pattern’. The consensus among herpetologists is that snakes with this pattern can be detected more distinctively and be perceived as more threatening [17]. Nevertheless, the results of the present study demonstrate the phenomenon of variability in such rapid snake detection by humans according to the postural variations of snakes to which the humans are exposed. Namely, the detection is more rapid when a typical striking posture is displayed by snakes. The fact strongly indicates the possibility that what has evolved to be the prototypical images of snakes arousing fear in humans would be close to those of snakes that are displaying a striking posture.

In perception of human infant images, too, a similar phenomenon has been reported, namely, that effects of infantile physical attributes are enhanced by such behavioral characteristics as clumsiness in the overall motion of infants [18]. When a striking posture is taken by snakes, they display their specific morphological characteristics as signals towards the presumptive signal receivers so that the receivers will categorize them as snakes as efficiently as possible, be threatened and withdraw. Consequently, the striking posture may have evolved in snakes in part to accentuate an attention-retting property. Such enhancement of the perceptual uniqueness and prominence of the basic physical design features of the snake may enable humans to more rapidly identify imminent danger in the form of snakes poised to strike [19]. Alternatively, it may be a manipulation by the snakes of the humans, i.e. an adaptation of the snakes. Which explanation is more plausible cannot be determined at the moment. Moreover, if one can find the presence of any particular snakes more provocative than others independent of pose, this would be important information. Indeed, it has been argued that the highly periodic pattern as well as the diamond-shape pattern of snake skins, which is unique among objects in nature, might be important cues to use when the whole sinusoidal form of snakes cannot be seen because it is occluded by natural objects [3], [20]. Apparently, these are issues to be investigated in the future.
